# OPTIMIZING CARE OF OLDER ADULTS BY USING 4MS AT A COMMUNITY HEALTH CENTER

**DOI:** 10.1093/geroni/igae098.3563

**Published:** 2024-12-31

**Authors:** Karla Tejada Arias, Elizabeth Hernandez, Jane Borrero De Jesus, Andrea Rink, Richard Marottoli, Jennifer Ouellet, Noelle Fry, Barry Wu

**Affiliations:** Fair Haven Community Health Care, New Haven, Connecticut, United States; Yale New Haven Hospital, New Haven, Connecticut, United States; Fair Haven Community Health Care, New Haven, Connecticut, United States; Yale University, New Haven, Connecticut, United States; Yale University School of Medicine, New Haven, Connecticut, United States; Yale University, New Haven, Connecticut, United States; Yale University, New Haven, Connecticut, United States; Yale University, New Haven, Connecticut, United States

## Abstract

Given the increasing needs of the aging population and limited availability of geriatricians, it is crucial for community healthcare centers to develop effective approaches for geriatric care. Fair Haven Community Health Care, implemented the 4Ms Framework of Age-Friendly Care, from the Institute for Healthcare Improvement for patients 65 and older starting July 2023 at two different clinical sites. We employed screening tools and interdisciplinary team including patient-access, medical assistants, nurses, clinicians and resident physicians, to evaluate cognitive health, mobility, and medication regimens, prioritizing patient-centered care. The Electronic Medical Record was enhanced to include fall risk and cognitive assessments. Feasibility was assessed through surveys. This intervention has been applied to 196 patients, 54% of whom identified as Hispanic, 29% white and 13% African American. A staff survey (N=23) revealed a 100% satisfaction rate, with no adverse effects on workflow. Overall visit times remained consistent, with rooming times for patients under the 4Ms framework increasing by only 11%, indicating no substantial impact on workflow efficiency. Additionally, patient satisfaction increased considerably, with patients reporting a greater sense of involvement in their healthcare decision-making process and felt their care was aligned with their priorities, demonstrating a successful transition to patient-centered care. Thus, we have demonstrated that the 4Ms framework can be integrated into standard care for older adults in a busy community health center. This successful integration highlights our ability to deliver evidence-based care and boost patient involvement, signaling a positive transition towards personalized, patient-centered healthcare delivery for this vulnerable demographic.

